# Invasive fungal tracheobronchitis in mechanically ventilated critically ill patients: underlying conditions, diagnosis, and outcomes

**DOI:** 10.1186/s13613-016-0230-9

**Published:** 2017-01-06

**Authors:** Chun-Yu Lin, Wei-Lun Liu, Che-Chia Chang, Hou-Tai Chang, Han-Chung Hu, Kuo-chin Kao, Ning-Hung Chen, Ying-Jen Chen, Cheng-Ta Yang, Chung-Chi Huang, George Dimopoulos

**Affiliations:** 1 Department of General Medicine and Geriatrics, Chang Gung Memorial Hospital at Linkou, Taoyuan, Taiwan; 2 Department of Pulmonary and Critical Care Medicine, Chang Gung Memorial Hospital at Linkou, Taoyuan, Taiwan; 3 College of Medicine, Chang Gung University, Taoyuan, Taiwan; 4 Department of Intensive Care Medicine, Chi Mei Medical Center, Liouying, Tainan, Taiwan; 5 College of Health Sciences, Graduate Institute of Medical Sciences, Chang Jung Christian University, Tainan, Taiwan; 6 College of Medicine, Fu Jen Catholic University, New Taipei, Taiwan; 7 Department of Pulmonary and Critical Care Medicine, Chang Gung Memorial Hospital at Chiayi, Chiayi, Taiwan; 8 Department of Critical Care Medicine, Far Eastern Memorial Hospital, New Taipei City, Taiwan; 9 Department of Critical Care, ATTIKON University Hospital, University of Athens, Medical School, Athens, Greece

**Keywords:** Invasive fungal tracheobronchitis, Aspergillosis, Mucormycosis, Critical care, Outcome

## Abstract

**Background:**

Invasive fungal tracheobronchitis (IFT) is a severe form of pulmonary fungal infection that is not limited to immunocompromised patients. Although respiratory failure is a crucial predictor of death, information regarding IFT in critically ill patients is limited.

**Methods:**

In this retrospective, multicenter, observational study, we enrolled adults diagnosed as having IFT who had been admitted to the intensive care unit between January 2007 and December 2015. Their demographics, clinical imaging data, bronchoscopic and histopathological findings, and outcomes were recorded.

**Results:**

This study included 31 patients who had been diagnosed as having IFT, comprising 24 men and 7 women with a mean age of 64.7 ± 13.7 years. All patients developed respiratory failure and received mechanical ventilation before diagnosis. Eighteen (58.1%) patients had diabetes mellitus, and 12 (38.7%) had chronic lung disease. Four (12.9%) patients had hematologic disease, and none of the patients had neutropenia. Twenty-five (80.6%) patients were diagnosed as having proven IFT, and the remaining patients had probable IFT. *Aspergillus* spp. (61.3%) were the most common pathogenic species, followed by Mucorales (25.8%) and *Candida* spp. (6.5%). The diagnoses in six (19.4%) patients were confirmed only through bronchial biopsy and histopathological examination, whereas their cultures of bronchoalveolar lavage fluid were negative for fungi. The overall in-hospital mortality rate was 93.5%.

**Conclusions:**

IFT in critically ill patients results in a high mortality rate. Diabetes mellitus is the most prevalent underlying disease, followed by chronic lung disease. In addition to *Aspergillus* spp., Mucorales is another crucial pathogenic species. Bronchial lesion biopsy is the key diagnostic strategy.

## Background

Invasive fungal disease is a life-threatening disease that mostly occurs in immunocompromised patients. The incidence of pulmonary fungal infection has dramatically increased in recent years [1]. *Aspergillus* spp. is the most common pathogenic species among pulmonary fungal infection [2, 3]. The overall mortality rate of invasive aspergillosis is approximately 50% [4–6]. Moreover, the frequency of invasive fungal infections caused by non-*Aspergillus* filamentous fungi is also increasing, and these infections are associated with devastating outcomes similar to that of invasive aspergillosis [5]. In addition to patients with conventional risk factors including neutropenia and those who have undergone stem cell transplantations, patients with chronic obstructive pulmonary disease, chronic renal failure, and liver cirrhosis may develop invasive fungal infections [2, 7, 8]. Critically ill patients who are admitted to intensive care units (ICUs) have been increasingly recognized as a population at a particularly high risk of pulmonary fungal infection [2]. Moreover, invasive aspergillosis in critically ill patients without malignancy who receive mechanical ventilation results in very poor outcomes and a mortality rate of 90% [9].

Invasive fungal tracheobronchitis (IFT) is a rare but severe form of pulmonary fungal infection that has been increasingly observed in critically ill patients [2, 10]. Diagnosing IFT is considerably difficult because of the nonspecific clinical manifestations and the low yields in microbiological tests [2, 11, 12]. The mortality rate of IFT caused by different fungi varies from 20 to 80% [11, 13–16]. Patients with *Aspergillus* tracheobronchitis who have developed acute respiratory failure exhibit substantially poorer outcomes than those without respiratory distress do (mortality rate 69.2–93.8 vs. 25–32.8%) [11, 15, 16]. Moreover, ICU admission is a strong predictor of death in patients with non-*Aspergillus* mold invasive infections [5]. However, information regarding IFT in critically ill patients is limited.

The aim of the current study is to evaluate the diagnostic approach and the outcomes of IFT in critically ill patients.

## Methods

### Study design and subjects

In this retrospective, multicenter, observational study, we included critically ill adult patients with IFT who had been admitted to medical ICUs between January 2007 and December 2015 at the Linkou and Chiayi branches of Chang Gung Memorial Hospital, Far Eastern Memorial Hospital, and the Liouying branch of Chi Mei Medical Center. This study was approved by the institutional review boards of Chang Gung Memorial Hospital (CGMH 104-7452B). The patients were classified as having proven or probable IFT by using the revised definitions for invasive fungal infections from the European Organization for the Research and Treatment of Cancer/Mycosis Study Group (EORTC/MSG) [17]. Histopathology was used to diagnose proven IFT. Probable IFT refers to the presence of positive cultures for fungal species from bronchoalveolar lavage (BAL) specimens accompanied by tracheobronchitis. All patients underwent fiberoptic bronchoscopy. From the bronchoscopic findings, IFT was classified into pseudomembranous, ulcerative, or obstructive forms according to Denning’s classification [18]. The patients’ demographic data; underlying diseases; clinical presentation; disease severity; laboratory parameters; bronchoscopic, microbiological, and histopathological findings; medications; and outcomes were recorded.

Overall in-hospital mortality was assessed. If the study patients were alive, survival was recorded until the date they were lost to follow-up or the date the study concluded. Because of the retrospective, observational nature of this study and the lack of any modification in the general management of the patients, the need for informed consent was waived.

### Statistical analyses

All statistical analyses were performed using GraphPad Prism statistical software (GraphPad Prism, version 5.01). The categorical variables are presented as counts (percentages), and the continuous variables are presented as the means ± standard deviations.

## Results

**Fig. 1:**
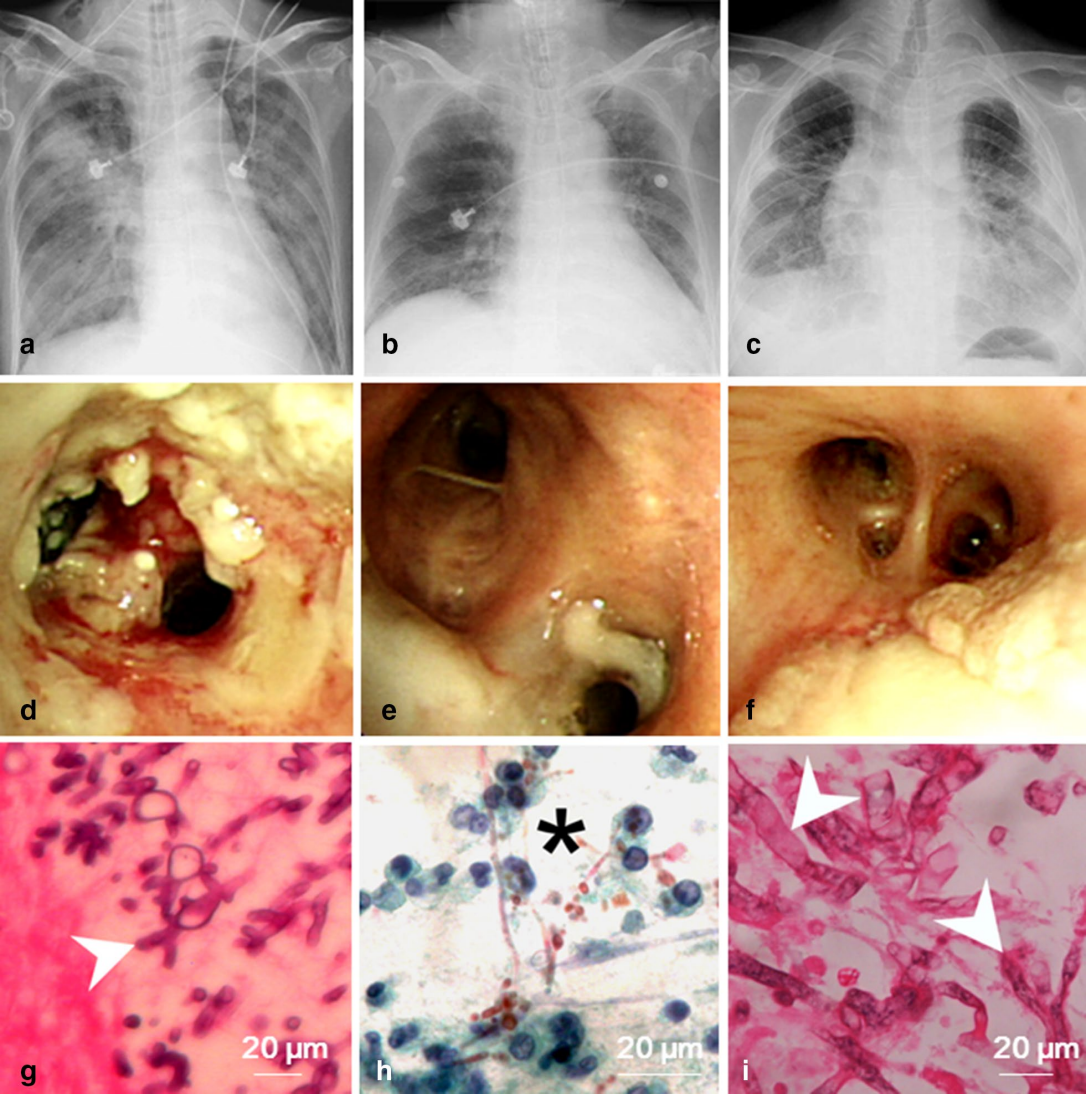
Chest radiograph and bronchoscopic and histological examinations of IFT. **a** Invasive Aspergillus tracheobronchitis. **b** Invasive Candida tracheobronchitis. **c** Mucorales-related invasive tracheobronchitis. **d** Bronchoscopic view of invasive Aspergillus tracheobronchitis. **e** Bronchoscopic view of invasive Candida tracheobronchitis. **f** Bronchoscopic view of Mucorales-related invasive tracheobronchitis. **g** Histopathological examination revealed septate fungal hyphae branching at a 45° angle (*arrowhead*), which is characteristic of *Aspergillus* spp. (magnification: 400×). **h** Histopathological examination revealed yeast cells and pseudohyphae (*star*), which are characteristic of *Candida* spp. (magnification: 400×). **i** Histopathological examination revealed broad, thin-walled, non-septate hyphae, which are characteristic of Mucormycete (*left arrowhead*) and the other septate fungal hyphae branching at a sharp angle, which are characteristic of *Aspergillus* spp. (*right arrowhead*) (magnification: 400×)

This study included 31 critically ill patients who had been diagnosed as having IFT, comprising 24 men and 7 women with a mean age of 64.7 ± 13.7 years. Table 1 summarizes the demographics and underlying conditions of the patients who were hospitalized in the medical ICU for IFT. Thirty (96.8%) patients had underlying diseases. Only one patient, who was a light smoker, had no medical history. Diabetes mellitus (DM; 18 patients, [58.1%]) was the most predominant underlying condition in the IFT patients. The median HbA1c level was 8.1% (5.4–13%). Five patients were newly diagnosed as having DM. Nine patients were taking oral anti-diabetic agents. Four patients had received insulin therapy. Three of these DM patients had proteinuria and chronic renal disease. Chronic lung disease (12 patients [38.7%]) was the second most predominant underlying disease. Four patients (12.9%) had solid organ tumors, and four (12.9%) had hematologic disease. None of these IFT patients had chronic renal failure or neutropenia, and none had undergone solid organ transplantations. Moreover, 17 patients (54.8%) had received systemic steroid treatment before diagnosis. Three patients (9.7%) developed IFT after being diagnosed as having H1N1 pneumonia. The overall in-hospital mortality rate was 93.5% (29 patients). The median time spent in the ICU before diagnosis was 5 days (0–19 days). The median length of ICU stay was 14 days (2–85 days). The median survival time after diagnosis was 10 days (0–85 days). Table 2 summarizes the clinical manifestations of the critically ill patients with IFT. The mean Acute Physiology and Chronic Health Evaluation II (APACHE II) score on ICU admission was 23.1 ± 10.4. Because of respiratory failure, all patients received mechanical ventilation before diagnosis. Furthermore, 21 patients (67.7%) underwent computed tomography (CT). Consolidation was the most frequent finding (19 patients [61.3%]). Only one patient exhibited the air crescent sign on CT, and none of the patients had the halo sign. All patients underwent bronchoscopies and BAL. Moreover, 27 and 12 patients (87.1 and 38.7%) had the pseudomembranous and ulcerative forms of IFT, respectively. The obstructive form (4 patients [12.9%]) was the least frequent form of IFT (Table 2). Bronchial biopsy was performed in 27 patients (87%). Biopsy was not performed in the remaining four patients (12.9%) because of the extremely low platelet count (<5000 per mm^3^) or severe hypoxemia under ventilator support (fraction of inspired oxygen 100%). None of the patients had developed massive hemoptysis or hypoxemia after the bronchial biopsy. Figure 1 illustrates the distinct histological images and indistinguishable bronchoscopic and radiographic findings of IFT caused by different fungal species. The mortality rate did not differ significantly between the patients with proven and probable IFT (96% [24 of 25] vs. 83.3% [5 of 6]; *p* = 0.35).

**Table 1:** Demographics and underlying conditions of 31 patients with invasive fungal tracheobronchitis

Variable	No. of patients (%)
Age, years (mean ± SD)	64.7 ± 13.7
Gender, male	24 (77.4)
Current/ex-smoker	15 (48.4)
Underlying disease	30 (96.8)
DM	18 (58.1)
Chronic lung disease	12 (38.7)
COPD/asthma	8 (25.8)
Old TB	2 (6.5)
Bronchiectasis	2 (6.5)
Solid organ cancer	4 (12.9)
Hematologic disease	4 (12.9)
Liver cirrhosis	3 (9.7)
Systemic steroids before diagnosis	17 (54.8)
Duration of steroids before ICU admission, day, median (IQR)	49 (14–90)
Daily dosage of steroids, mg, median (IQR)	50 (29–71)
Inhaled corticosteroids before diagnosis	3 (9.7)
H1N1 infection before diagnosis	3 (9.7)

*SD* standard deviation, *DM* diabetes mellitus, *COPD* chronic obstructive pulmonary disease, *TB* tuberculosis, *ICU* intensive care unit, *IQR* interquartile range

**Table 2:** Clinical manifestations of 31 patients with invasive fungal tracheobronchitis

Variable	No. of patients (%)
APACHE II score on ICU admission, mean ± SD	23.1 ± 10.4
AKI requiring RRT	14 (45.2)
RF before diagnosis reached	31 (100)
Time in the ICU before diagnosis, days (IQR)	5 (1.8–8)
Length of ICU stay, days (IQR)	14 (8–27)
Concurrent bacterial sepsis	18 (58.1)
Parenchymal involvement	31 (100)
CT scan	21 (67.7)
Consolidation	19 (61.3)
Cavitation	4 (12.9)
Air crescent sign	1 (3.2)
Bronchoscopic classification	
Pseudomembranous	27 (87.1)
Ulcerative	12 (38.7)
Obstructive	4 (12.9)
Diagnosis of IFT	
Proven	25 (80.6)
Probable	6 (19.4)
Pathogen	
*Aspergillus* spp.	19 (61.3)
*Aspergillus fumigatus*	11 (35.4)
*Aspergillus flavus*	2 (6.5)
*Aspergillus terrus*	1 (3.2)
Undifferentiated *Aspergillus* species	5 (16.1)
*Mucorales*	8 (25.8)
*Candida* spp.	2 (6.5)
Undifferentiated mold	2 (6.5)
BAL fungal culture	
Positive	25 (80.6)
Negative	6 (19.4)
Galactomannan level in *Aspergillus* tracheobronchitis	
Serum (index)	3.44 ± 2.8
BAL (index)	4.87 ± 2.6
Antifungal therapy	29 (93.5)
Voriconazole	15 (48.4)
Echinocandin	19 (61.3)
Combination therapy	8 (25.8)

*APACHE II* Acute Physiology and Chronic Health Evaluation II, *ICU* intensive care unit, *SD* standard deviation, *AKI* acute kidney injury, *RRT* renal replacement therapy, *RF* respiratory failure, *IQR* interquartile range, *CT* computed tomography, *IFT* invasive fungal tracheobronchitis, *BAL* bronchoalveolar lavage

In this study, *Aspergillus* spp. was found to be the most common pathogenic species of IFT (19 patients [61.3%]), followed by Mucorales (8 patients [25.8%]). *Candida* spp. was the causative pathogen of IFT in two patients (6.5%). A further two patients were infected by undifferentiated mold (Table 2). The galactomannan antigen levels were evaluated in 16 of the 19 patients with *Aspergillus* tracheobronchitis and in 4 of the 12 patients with non-*Aspergillus*-related fungal tracheobronchitis. In patients with *Aspergillus* tracheobronchitis, the galactomannan antigen levels in the serum and BAL were 3.44 ± 2.8 and 4.87 ± 2.6, respectively. In patients with non-*Aspergillus*-related fungal tracheobronchitis, the galactomannan antigen levels were less than 0.5. The two survivors were infected by *Aspergillus* spp. and undifferentiated mold. The mortality rate did not differ among patients with IFT caused by different fungal species. Only nine patients (29%) had positive fungal culture results for lower respiratory tract specimens before bronchoscopy. Six patients (19.4%) had negative fungal culture results for BAL fluid after bronchoscopy. They were diagnosed through histopathology of bronchial biopsy specimens.

## Discussion

To the best of our knowledge, this study was the largest series of IFT in mechanically ventilated critically ill patients. The main findings of our study are as follows. First, 80.6% of the patients had proven IFT, whereas the remaining patients (19.4%) had probable IFT. Second, DM was the most frequent predominant underlying condition in our IFT patients (58.1%), followed by chronic lung disease (38.7%). Third, less than 15% of the patients had a hematologic disease. Fourth, none of the patients had neutropenia or had undergone solid organ transplantations. Fifth, the mortality rate was 93.5%. All patients developed acute respiratory failure before diagnosis and were admitted to the ICU with high APACHE II scores (23.1 ± 10.4).

Invasive fungal infection was reported to mainly affect immunocompromised patients, particularly those with hematologic malignancy [17]. However, *Aspergillus* spp. has recently been shown to cause invasive fungal disease in patients with chronic lung disease and critically ill patients with liver cirrhosis [7, 19–24]. This is mainly attributed to the administration of broad spectrum antibiotics, corticosteroids, and immunoparalysis related to sepsis [2, 22, 25–27]. In a cohort study of 156 patients with *Aspergillus* tracheobronchitis, Fernández-Ruiz et al. [15] reported that 6.5% of them had DM, 23.7% received mechanical ventilation because of respiratory failure, most patients were immunocompromised, and the mortality rate was 39.1%. Karnak et al. [13] reviewed 228 patients with endobronchial fungal disease and found that 54% of them were immunocompromised, and 11% had DM; the mortality rate was 52% in patients with endobronchial mucormycosis. In our cohort, DM was the most prevalent underlying condition (58.1%), whereas only four patients (12.9%) had hematologic disease, and none of the patients were neutropenic. Numerous studies have reported that acute respiratory failure and ICU admission are crucial prognostic factors for invasive fungal disease [5, 15, 27–30]. In the current study, we found that patients with IFT who developed respiratory failure exhibited high mortality. He et al. [14] reported that patients with *Aspergillus* tracheobronchitis and the involvement in parenchyma demonstrated higher mortality, suggesting that *Aspergillus* tracheobronchitis is an early stage of invasive pulmonary aspergillosis. By contrast, Patterson and Strek [2] argued that tracheobronchitis is a form of invasive pulmonary aspergillosis and is associated with poor outcomes because of delayed diagnosis. Furthermore, Karnak et al. [13] also found that 7–20% of patients with invasive pulmonary aspergillosis simultaneously manifested fatal tracheobronchial involvement. The overall mortality of invasive fungal infection is approximately 50% [5, 6], and the mortalities of endobronchial fungal disease caused by different fungi range widely from 20 to 80% [11, 13–16]. In our cohort, the overall in-hospital mortality rate was 93.5% even though none of the patients had neutropenia or immunosuppression. Cornillet et al. suggested that among non-neutropenic patients with invasive aspergillosis, the nonspecific symptoms and the difficulty in diagnosis lead to suboptimal management and the late administration of treatment. This delayed administration of treatment results in a higher mortality in non-neutropenic patients than in neutropenic patients (89 vs. 60%) and may explain the high mortality observed in our cohort [24, 31].

Diagnosing IFT is considerably difficult because of the nonspecific clinical manifestations and the lack of a diagnostic tool to distinguish colonization from infection [2, 12, 32, 33]. Radiological findings are usually nonspecific in patients with aspergillosis. The typical halo and air crescent signs on CT images of the lungs are associated with low sensitivity for invasive fungal infection and are rarely observed in non-neutropenic patients [12, 21]. In the current study, the air crescent sign was detected on the CT scan of only one patient. The galactomannan antigen levels in BAL fluid are useful for diagnosing invasive pulmonary aspergillosis [34]. However, in addition to *Aspergillus spp.*, other fungi may cause invasive tracheobronchitis without affecting the galactomannan antigen levels [13]. Invasive aspergillosis is the most frequent invasive fungal infection among hematologic patients (59.2–74.3%), followed by invasive fungal infections caused by Mucorales (7.2–13.9%) [35, 36]. Karnak et al. [13] also found that *Aspergillus* spp. is the most common pathogenic species of endobronchial fungal disease (53%), and Mucorales accounted for only 13.4%. In the current study, *Aspergillus* spp. was the most common pathogenic species (61.3%), followed by Mucorales (25.8%).

Considering the increasing incidence of invasive aspergillosis in critically ill patients and the strict host criteria in the revised definitions for invasive fungal disease from the EORTC/MSG [17], Blot et al. [37] validated a clinical algorithm to diagnose invasive pulmonary aspergillosis in the ICU. They proposed *Aspergillus*-positive lower respiratory tract specimen culture as the entry criterion and that physicians should increasingly consider the possibility of invasive pulmonary aspergillosis [20, 22, 37]. However, the rate of positive fungal culture results for BAL fluid is only 25–77% and can further decrease after antifungal therapy [13, 31, 32]. In our patients, less than 30% had positive cultures for fungi before bronchoscopy, and 20% had negative BAL fluid cultures after bronchoscopy. The incidence of IFT may be underestimated if the diagnosis is based only on positive cultures. Hence, we believe that bronchoscopy should be the main diagnostic approach for IFT.

In 1995, Denning proposed three forms of *Aspergillus* tracheobronchitis, pseudomembranous, obstructive, and ulcerative [18]. Thereafter, Karnak et al. [13] demonstrated that endobronchial fungal disease can be caused by six different fungi. The origins of invasive fungal diseases are difficult to differentiate on the basis of radiographic or bronchoscopic findings (Fig. 1). Direct microscopy of tracheobronchial specimens is essential for observing fungal morphology, enabling a presumptive diagnosis of IFT and earlier administration of antifungal treatment [12]. Transbronchial lung biopsy or bronchial aspiration is relatively risky in patients with angioinvasive fungal infection, particularly in patients with coagulopathy or thrombocytopenia [26, 38]. By contrast, the biopsy of bronchial lesions is less invasive and relatively safe; biopsy exhibits a higher sensitivity than BAL fluid culture alone [14, 16]. In our study, all patients underwent bronchoscopy and BAL cultures and 25 underwent bronchial biopsy. None of the patients developed massive hemoptysis or hypoxemia after the procedure.

The present study had some limitations. First, this study was retrospective. Second, this study included only cases with proven and probable IFT; thus, some of the possible cases may have been overlooked, and the outcomes may have been underestimated.

## Conclusion

IFT in mechanically ventilated critically ill patients is a devastating disease irrespective of the host’s immune status. DM is the most prevalent underlying disease, followed by chronic lung disease. The mortality rate is very high in IFT patients who develop respiratory failure. In addition to *Aspergillus* spp. (61.3%), Mucorales is another crucial pathogenic species (25.8%). Early diagnosis of IFT is based on bronchoscopy. Bronchoscopy with the biopsy of bronchial lesions should be the preferred diagnostic strategy. Large, prospective studies are urgently required to improve the outcome of IFT in critically ill patients.

## Abbreviations

IFT: invasive fungal tracheobronchitis
ICU: intensive care unit
BAL: bronchoalveolar lavage
COPD: chronic obstructive pulmonary disease
APACHE II: Acute Physiology and Chronic Health Evaluation II
CT: computed tomography
